# Empirical Validation of a Streamlined Three-Repetition Squat Protocol Using MAI Motion

**DOI:** 10.7759/cureus.115292

**Published:** 2026-08-27

**Authors:** Tanvi Verma, Mathew George, Yan Wen, Paul Lee

**Affiliations:** 1 Orthopaedics and Rehabilitation, MSK Doctors and Associates, Grantham, GBR; 2 Orthopaedics, University of Leicester, Leicester, GBR; 3 Research, MSK Doctors, Sleaford, GBR; 4 Trauma and Orthopaedics, MSK Doctors, Sleaford, GBR

**Keywords:** digital healthcare, kinematics, knee biomechanics, markerless, motion capture, movement analysis

## Abstract

MAI Motion is a motion capture system designed to assess lower limb biomechanics during functional movements such as the squat. Determining how many repetitions are needed to obtain consistent and comparable kinematic measurements is critical for balancing data quality and participant burden. This study evaluates whether three repetitions (3×) of the squat test provide comparable kinematic data to five repetitions (5×). 3D videos of healthy adult participants (n=10) performing 5× squat movements were captured using MAI Motion. Primary measurements were the mean values of each joint angle and the coefficient of variation (CV). Statistical comparisons (paired t-tests or non-parametric equivalents) determined that differences in mean values or CV existed between 3× and 5×. Analysis revealed minimal differences in mean angles between 3× and 5×. Variability, assessed via CV, showed no clinically meaningful differences (p<0.05). Although ankle angle and knee and hip abduction had higher CV values than other metrics, 3× and 5× performed similarly. Participants reported that 3× required less effort than 5×, implying a practical advantage for clinical or research settings. These findings validate that a 3× squat may capture biomechanical data comparable to a 5× protocol using the MAI Motion system. Reducing repetitions could possibly lessen participant fatigue while preserving the consistency of kinematic outputs, making 3× a pragmatic standard for most populations.

## Introduction

The integration of artificial intelligence (AI) in motion capture has gained significant attention across multiple fields, especially in healthcare and rehabilitation. However, the advancement of AI-based motion tracking systems employing markerless technology is emerging as a viable alternative to traditional methods that rely on physical markers and controlled laboratory environments. These advancements are particularly relevant in clinical settings where the use of markers may be impractical or invasive.

These AI-based investigative tools facilitate motion capture in diverse clinical domains, including orthopaedic clinics, physiotherapy, and rehabilitation, without the need for extensive physical setups [1]. The shift towards markerless motion capture is further supported by the lower cost, greater accessibility, and ability to perform whole-body kinematics tracking [2].

MAI Motion is a markerless digital platform that allows the analysis of everyday movements including the transition from a standing to squat position known as a full repetition squat [3,4]. The number of repetitions performed during assessment is a key methodological factor, as fewer repetitions may compromise measurement stability, whereas higher repetition protocols may reduce feasibility in clinical populations due to pain, fatigue, or movement limitation. Establishing whether a reduced repetition protocol can maintain consistent kinematic measurements is therefore critical for both methodological robustness and clinical implementation. The transition from a standing to a squat position involves a multifaceted process that requires a complex interplay of kinematic phases and biomechanical adjustments. The muscular mechanism of squat exercises is fundamental to understanding their effectiveness in strength training, rehabilitation, and overall function. Squats primarily engage various muscle groups in the lower extremities, employing a complex interplay of agonists, antagonists, and stabilisers. The primary muscles involved include the quadriceps, hamstrings, gluteus maximus, and, to a lesser extent, calves and core muscles that provide stabilisation [5].

Squat performance relies on the coordinated activation of key lower limb muscle groups, including the quadriceps, hamstrings, and gluteus maximus. The quadriceps act as the primary knee extensors during ascent, with greater activation at increased squat depths [6]. Meanwhile, the hamstrings contribute to controlled knee flexion and hip stabilisation, functioning eccentrically during descent to regulate movement and maintain alignment [7,8]. The gluteus maximus becomes increasingly activated both as a hip extensor and as a stabiliser of the pelvis and lumbar spine, especially at greater squat depths and loads [9]. In addition, coordinated engagement of core musculature, including the abdominal and spinal stabilisers, is essential for maintaining trunk control during the upward phase while maintaining postural integrity.

In addition to the primary movers, core musculature, including the abdominal and spinal stabilisers, contributes significantly to maintaining proper technique during squats. This is essential to avoid improper load distribution and maintain proper core activation essential for maintaining a strong and stable posture throughout the squat movement [10]. The integrated approach of recruiting not just the lower limbs but also the core during squat relies on the harmonious activation and coordination of these muscles which provides safety, minimising the risk of injury [11].

The squat test is primarily conducted for its ability to effectively indicate lower limb strength, functional capacity, and risk of falls, which are critical for maintaining independence in daily activities [12]. Its biomechanical complexity and established role in clinical and research assessment make it particularly suitable for evaluating lower limb kinematics using motion capture systems such as MAI Motion.

The squat test is a widely utilised and validated tool in clinical and research settings for assessing lower limb function, balance, and physical performance, particularly among older adults and individuals with musculoskeletal (MSK) impairments [13,14]. Squats play a significant role in motion capture due to their biomechanical complexity and the insights they provide into joint loading and overall kinematics during physical activities. Motion capture technology, often employed in conjunction with biomechanical analysis software, allows for the detailed assessment of how different squat variations and depths affect lower extremity mechanics. While various squat protocols exist, the five-repetition format (5× squat) is commonly adopted due to its balance between practicality and sensitivity in detecting functional deficits [15,16]. However, there is limited evidence defining the minimum number of repetitions required to achieve reliable kinematic measurements, particularly in clinical populations where tolerance to repeated movement may be restricted. This provides a rationale for comparing three and five repetitions in the present study.

However, in clinical practice, a significant proportion of individuals with chronic MSK conditions are unable to complete five full repetitions due to pain, fatigue, or movement limitations. This constraint presents a potential barrier to inclusive assessment and data acquirement, particularly when advanced kinematic analysis tools such as MAI Motion are employed.

To address this challenge, we hypothesised that three repetitions (3×) of the squat test may yield data of comparable kinematic outputs to the conventional five-repetition protocol. The aim of this study, therefore, was to systematically evaluate whether kinematic parameters captured via 3D MAI Motion during 3× squat trials could replicate those obtained from 5× trials. By directly comparing both protocols across a range of key biomechanical metrics, we sought to establish whether the shorter protocol offers a valid alternative, thereby enhancing accessibility without compromising the integrity of the diagnostic assessment. This was assessed using paired comparisons, with difference in means (DiM) and coefficient of variation (CV) used to evaluate consistency and variability between conditions.

## Materials and methods

This study was conducted at MSK House, MSK Doctors, Silk Willoughby, Sleaford, in accordance with Good Clinical Practice guidance ICH-GCP E6(R2) [17]. All participants provided informed consent prior to taking part.

Ten participants were included in the study (seven male and three female; mean age: 60±10.5 years (mean±SD); range: 42-73 years). Participants were recruited and screened using predefined eligibility criteria. Inclusion criteria were the ability to safely complete the squat test for five consecutive repetitions (5×). The primary exclusion criterion was the inability to complete the required five repetitions of the squat test. This study was designed as a pilot evaluation to assess the feasibility and preliminary comparative consistency of reduced repetition analysis relative to the conventional five-repetition approach.

Movement was recorded using a standard RGB camera (resolution: 1920×1080 pixels, frame rate: 30 frames per second). Each participant performed the squat test following a standardised protocol. Participants were instructed to begin in an upright standing position with both feet placed flat on the ground in a neutral alignment and positioned approximately shoulder-width apart, with their arms in a comfortable position and not used for external support. Participants were then instructed to squat "as low as possible" to complete a full movement cycle (upright standing to descent, reaching the lowest position, followed by return to upright standing). This was repeated for five consecutive squat cycles, performed at a self-selected pace with the aim of maintaining consistency across repetitions. All recordings were performed in a controlled indoor clinical environment with consistent lighting and a neutral background, free from visual obstruction. Prior to recording, each participant stood facing the camera while the camera position was adjusted to ensure full-body (head-to-toe) visibility within the frame, which is required for accurate markerless motion analysis, as incomplete visual capture prevents successful kinematic processing. Participants were then instructed to perform the squat protocol, and their movements were recorded and uploaded onto the MAI Motion platform for markerless kinematic analysis and extraction of joint kinematics.

Through the MAI Motion platform, kinematic parameters were derived for knee, hip, and ankle angles, knee and hip abduction, and measures of left/right (L/R) movement and core movement. Measurements were determined for each participant across either all five repetitions (5× dataset) or the first three repetitions (3× data subset) of the same recording session, enabling comparison of whether analysis based on three repetitions provides comparable outputs to analysis based on five repetitions.

Kinematic parameters were defined as follows. For joint angle and abduction measures (knee, hip, and ankle), impulse (+ve and −ve), as defined by the MAI Motion platform, represented the speed of change in degrees per second (°/s), impulse ratio was defined as the positive impulse divided by the negative impulse (+ve impulse/−ve impulse) and reported as a unitless value, and range represented the range of motion in degrees (°). For measures of L/R movement and core movement, impulse (+ve and −ve) represented the speed of change in metres per second (m/s), impulse ratio was defined as the positive impulse divided by the negative impulse and reported as a unitless value, and range represented the range of motion in metres (m).

To quantify variability across repeated squat cycles, the CV was calculated as $(\text{standard deviation/mean})\times100$ and reported as a percentage (%). CV thresholds were interpreted using commonly cited cut-offs, with values <10% considered "ideal" and 10-20% considered "acceptable". To compare differences between analyses based on five versus three repetitions, the DiM was calculated as (5× × 3×)/5× × 100 and reported as a percentage, where 5× represented the mean derived from five repetitions and 3× represented the mean derived from the first three repetitions only.

Statistical analyses were performed using Microsoft Excel (Microsoft 365, Microsoft Corporation, Redmond, Washington, United States) and GraphPad Prism (Version 10.4.1, GraphPad Software, Boston, Massachusetts, United States). Data distribution was assessed for normality using Shapiro-Wilk testing alongside visual inspection of distribution plots. Paired analyses were performed to compare outcomes derived from three versus five repetitions as comparative exploratory assessments of within-session consistency, with paired t-tests applied as appropriate for within-subject comparisons. Ordinary one-way ANOVA with Šídák's multiple comparisons tests was applied where multiple conditions were evaluated. Statistical significance was set at p<0.05, and specific statistical tests used for each comparison are provided in the corresponding figure legends.

## Results

This study employed 3D MAI Motion capture technology to analyse key kinematic parameters, including positive and negative impulse, impulse ratio, and range of motion across critical joint movements, or categories, such as knee angle, knee abduction, hip angle, hip abduction, ankle angle, as well as left/right and core movement. These parameters were assessed during a squat performed five times (5×) by 10 participants, providing a comprehensive evaluation of dynamic joint and body motion under varying biomechanical loads. The purpose of this evaluation was to determine whether 5× repeats are necessary or whether 3× repeats provide comparable data and results.

**Figure 1:**
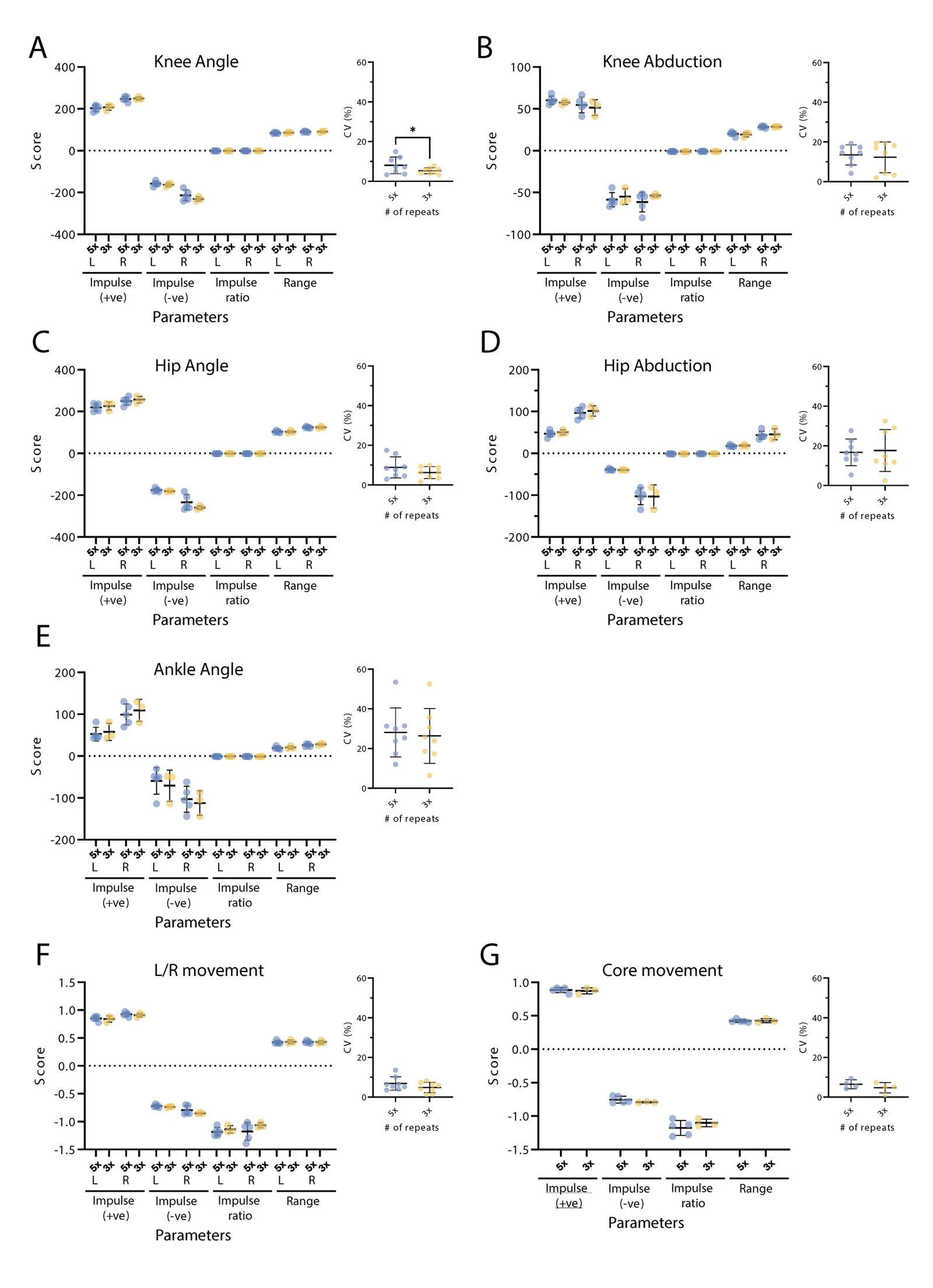
Overview of kinematic measurements from a participant performing a repeated squat test following the analysis of five or three repeats Measures for each parameter (impulse +ve, impulse −ve, impulse ratio, and range of motion (Range)) within each of the categories, (A) knee angle, (B) knee abduction, (C) hip angle, (D) hip abduction, (E) ankle angle, (F) L/R movement, and (G) core movement, were extracted from video of a participant performing the squat test five times captured using MAI Motion [3,4]. Each panel depicts parameters measured across all five repeats (5×, blue) or the first three repeats (3×, yellow), the mean±SD (black lines), and, in the insets, the coefficients of variance (CV (%)) for each parameter derived from 5× or 3× repeats. Left (L) and right (R) are shown for all measures except core. For panels A-E, units are impulse +ve and impulse −ve (o/s) and range (o). For panels F-G, units are impulse +ve and impulse −ve (m/s) and range (m). No significant differences were observed between the means (using ordinary one-way ANOVA followed by multiple comparisons) or CVs (using paired t-tests) of any parameter comparing 5× vs. 3×.

Figure 1 shows an overview of representative data from one participant. While there is considerable variation across the parameter scores within categories (e.g. when comparing left and right positive and negative impulse scores for knee angle (Figure 1A)) and across categories (when comparing the same parameters for knee, hip, and ankle angle (Figure 1A-1C)), there is relatively limited variation between scores derived from 5× vs. 3× for the same parameter, with means and standard deviations comparable. Consistent with this, there were no significant differences between the means for scores derived from 5× or 3× for the same parameter. Furthermore, analysis of the CV across the scores (Figure 1A-1G; see insets) showed comparable CVs for 5× and 3×, with no statistically significant differences and typically in the range of 10-20%, values considered ideal to acceptable in this context. This level of variability indicates stable kinematic behaviour across repeated squat cycles, with comparable consistency between three and five repetitions.

**Figure 2:**
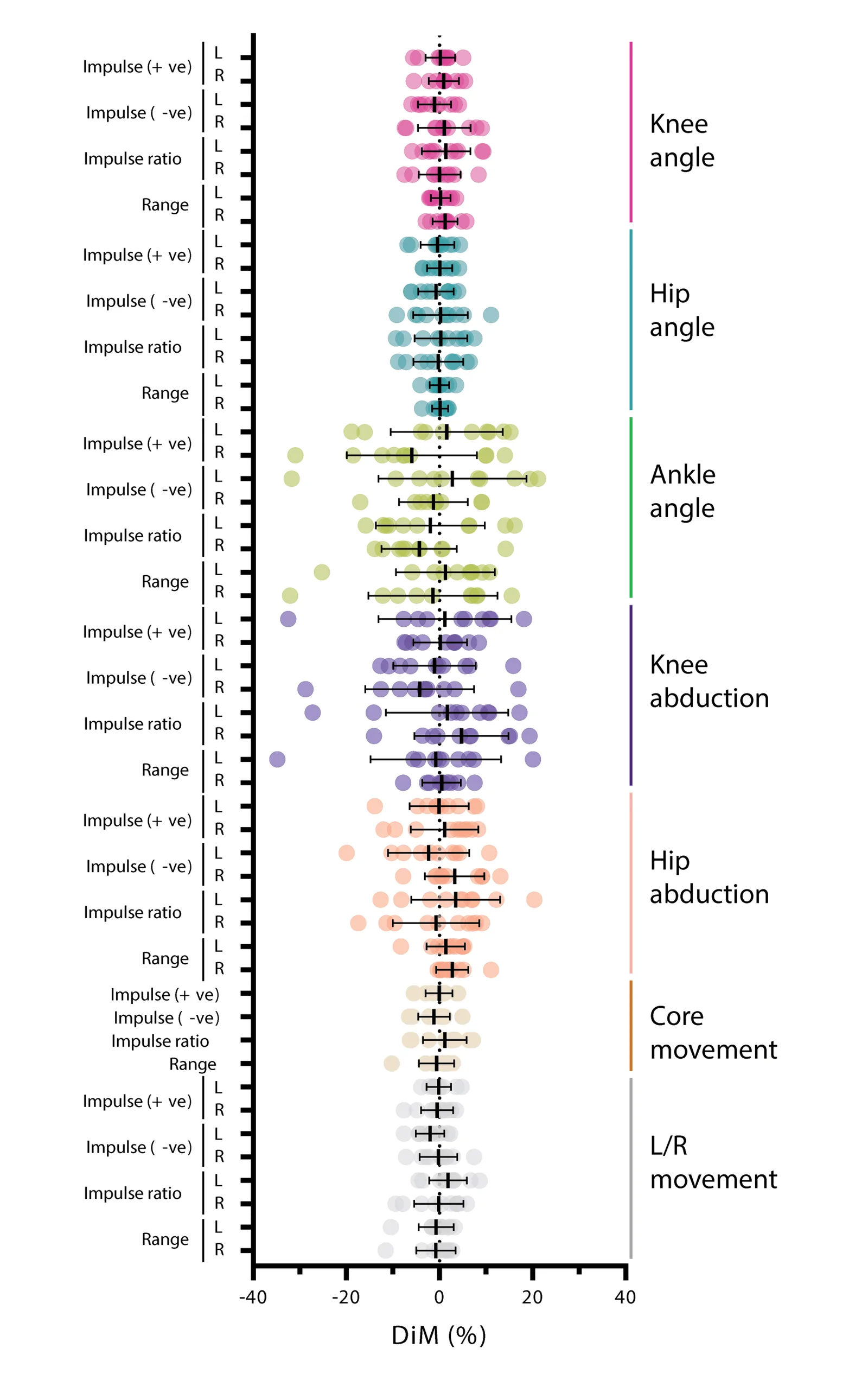
Difference in means (DiMs) derived from the analysis of 5× or 3× repeats of the squat test grouped by parameter DiMs for each parameter were derived by establishing the difference between means calculated from 5× or 3× repeats for each participant (n=10) and are presented as % of 5× mean. Mean±SD (black lines) of the DiM for each parameter are shown.

Next, we went on to analyse the DiMs to establish whether there were any significant differences between the results generated from the analysis of 5× or 3× repeats from all 10 participants (Figure 2). There were no significant differences between 5× and 3×, with DiMs for most parameters typically ranging between 0% and 10%, indicating good consistency between 5× and 3× repeats (as illustrated by all the mean DiMs being less than 10%). Higher values, up to 30%, were seen for some individual DiMs (which represent the DiM for that parameter, for one participant), most notably impulse ratio in knee angle, ankle angle, and hip abduction (Figure 2). Similar results were seen when data were grouped by participant (see Appendices). Collectively, the absence of any statistically significant differences between the DiMs for 5× and 3× and the consistency between measures suggests that analysis of data from 3× repeats generates results comparable to those from 5× repeats.

**Figure 3:**
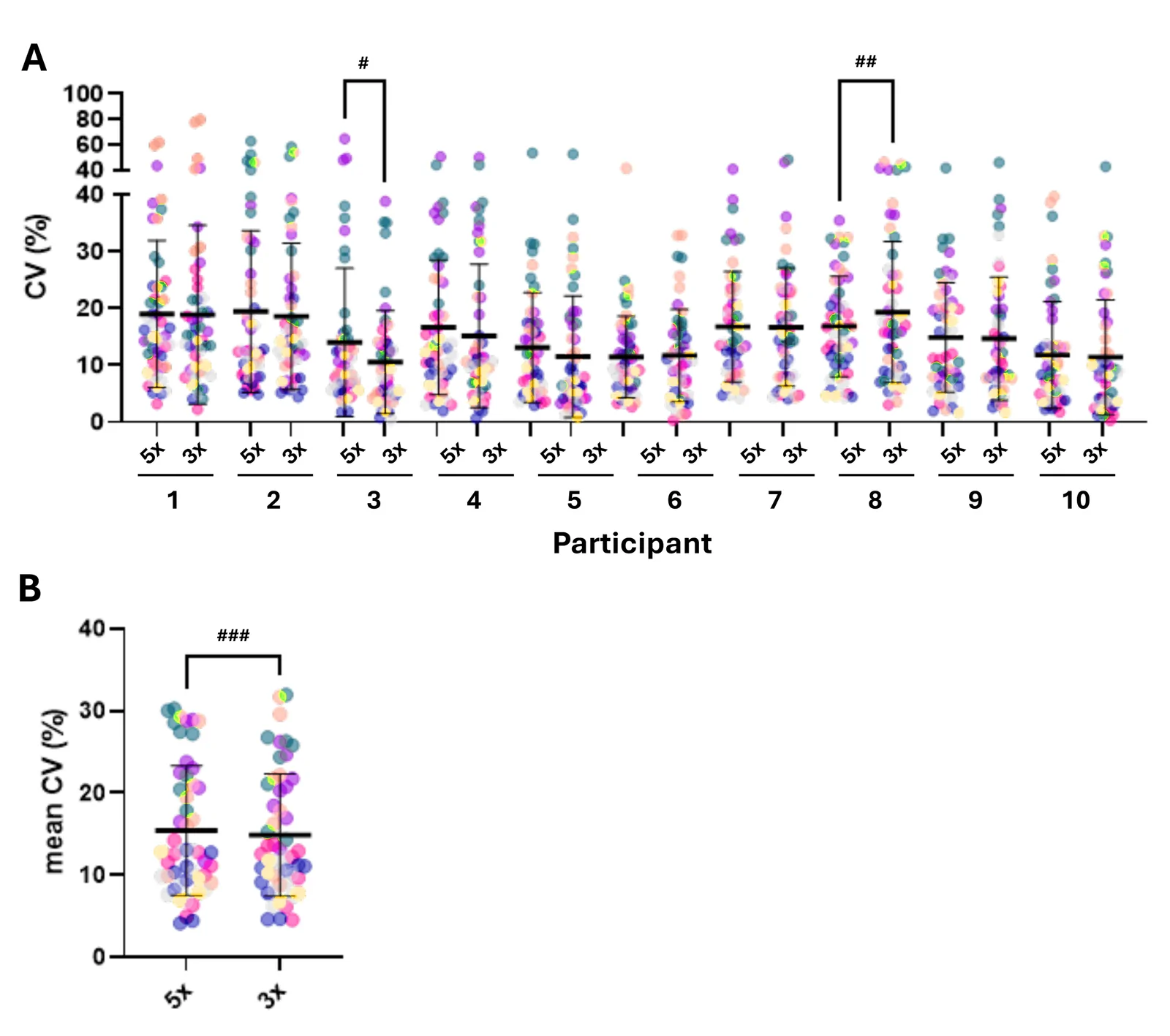
Coefficient of variance (CV) derived from the analysis of 5× or 3× repeats of the squat test grouped by participant (A) CVs for each parameter (describing knee angle (pink), knee abduction (purple), hip angle (blue), hip abduction (tan), ankle angle (teal), L/R movement (grey), and core movement (yellow)) were derived by comparing the CVs from 5× vs. 3× repeats for each participant (1-10). Mean±SD (black lines) are shown. Statistical significance between 5× and 3× for each participant was determined using repeated measures one-way ANOVA followed by Šídák's multiple comparisons tests (^#^p<0.025; ^##^p<0.002). (B) Mean CVs for each parameter across all 10 participants were calculated using data from 5× or 3× repeats. Mean±SD (black lines) are shown. Statistical significance between 5× and 3× was determined using a paired t-test (^###^p=0.0001).

**Figure 4:**
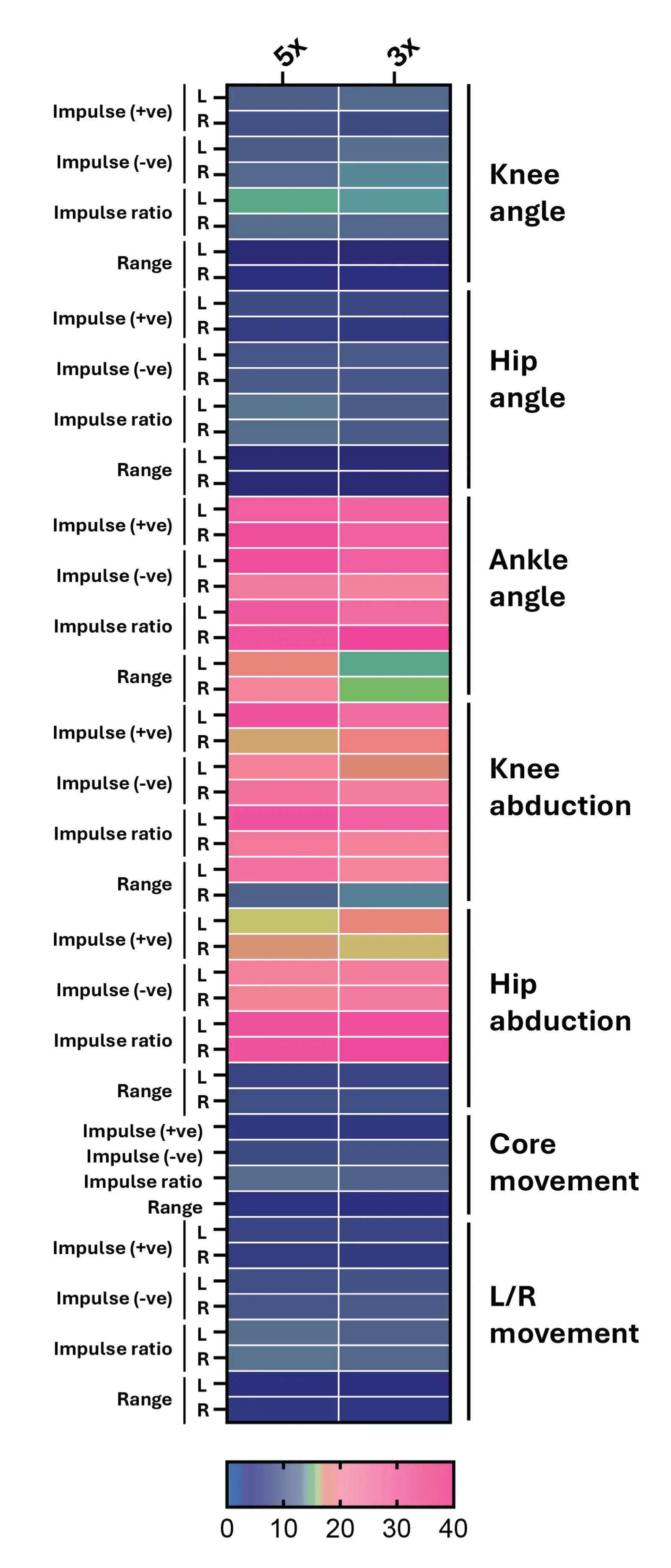
Summary of coefficients of variance (CVs) derived from the analysis of 5× or 3× repeats of the squat test grouped by parameter Mean CVs for each parameter from all 10 participants are represented using a colour scale ranging from blue (ideal/low CV), to amber (acceptable/intermediate CV), to pink (poor/high CV). Difference in colour for a parameter between 5× and 3× denotes differences between means of CVs for that parameter (e.g. 5× cf 3× for impulse −ve, L & R, knee angle). Similarly, where colours differ between the same parameter across different categories denotes differences between means of CVs for that parameter (e.g. impulse +ve and impulse −ve for knee angle cf ankle angle).

To investigate this further, we analysed the CVs generated from the 5× or 3× repeats from all participants (Figure 3). While most CV values were below 20%, in some instances, higher values around 60-70% were evident (e.g. participants 2 and 7, knee angle (pink) and hip abduction (tan), Figure 3A). Importantly, where such high values were found, they were present in both the 5× and 3× repeats. Statistically significant differences were revealed when comparing the CVs between 5× and 3× for participants 2, 6, and 7. For participants 2 and 7, the CVs were significantly lower in the 3× compared to 5× (mean±SD: 14.91±10.73 vs. 18.69±12.72 and 17.07±14.54 vs. 20.13±13.51; p<0.025), whereas this difference was reversed for participant 6 (mean±SD: 18.03±12.91 vs. 15.44±11.29; p<0.002; Figure 3A). Further statistical analysis of the mean CVs for each parameter (generated by calculating the mean CV for each parameter, itself derived by combining the mean CV for that parameter per participant) also showed a modest but significantly lower mean CV for 3× vs. 5× (mean±SD: 15.64±7.02 vs. 16.73±7.27; p<0.0001; Figure 3B). This is presented more qualitatively in Figure 4, which shows a comparison of the mean CV derived from 5× or 3× repeats for every parameter allowing the visual identification of variation between parameters and across categories as exemplified by differences in parameters across ankle angle and knee and hip abduction (Figure 4).

Overall, these quantitative and qualitative comparisons indicate that results generated from 3× are highly comparable to results from 5×.

## Discussion

This study aimed to evaluate whether the kinematic data generated by the MAI Motion system from participants performing the squat test over three repetitions was comparable to the data obtained from five repetitions. Existing studies evaluating markerless motion capture systems have primarily focused on reliability across sessions, tasks, or systems, often reporting measures such as intraclass correlation coefficients and standard error of measurement. However, there is considerable variability in testing protocols, and the optimal number of repetitions required to achieve stable kinematic outputs has not been clearly defined. The present study addresses this gap by directly comparing outcomes derived from three and five repetitions within a consistent testing framework.

The results indicate a high degree of consistency between results generated by the analysis of 3× or 5× repeats across multiple parameters, including positive and negative impulse, impulse ratio, and range of motion evaluated across key joint categories. Statistical and qualitative analysis demonstrated that results from 3× were broadly comparable to those from 5×. These findings support the potential use of a shorter 3× protocol, suggesting that it may be applied without substantial alteration in observed kinematic outputs or diagnostic benefit of the data.

Statistical analysis of results generated by the investigation of data from 5× or 3× repeats, including comparison of DiMs and CVs, showed a lack of significant differences between mean values or the levels of variability of all parameters when considered individually. Significant differences in variability were only observed when comparing grouped data, namely, the mean variation across CVs for all 52 parameters in three out of 10 participants [2] and the "meta-mean" of the CVs for each parameter, with the analysis of 3× repeats generating significantly less variability than that of 5×.

These findings are consistent with prior research in functional testing, indicating that beyond a modest number of repetitions, additional cycles yield diminishing returns on measurement precision. This likely reflects the effects of participant fatigue due to the increased number of repetitions, potentially resulting in greater variability in neuromuscular control, posture, and/or movement specifically across the fourth and fifth repeats. While further analysis would be required to address this more formally, previous studies have reported that increased fatigue can lead to adaptations in movement [18,19], giving rise to changes in biomechanical parameters [20]. Based on these observations and the results presented here, it seems reasonable to propose that 3× repeats may be considered as an alternative to reduce fatigue-related variability while maintaining the consistency of kinematic outputs, although further validation in larger cohorts is required.

From a diagnostic perspective, the opportunity to effectively capture movement dynamics using digital platforms such as MAI Motion has real-world applicability in MSK healthcare. For example, for individuals who are frail or exhibit considerable mobility impairments or chronic MSK conditions, the increased accessibility afforded through remote evaluation, combined with the adoption of a shorter protocol, will make assessments less demanding and more inclusive. Furthermore, many individuals undergoing rehabilitation find it challenging to complete five repetitions in a single session [21], making the 3× test a more realistic and inclusive option.

Limitations of this study are the relatively small number of participants, the limited diversity within the cohort, and the inclusion of analysis of only one functional task. As such, these findings should be interpreted as preliminary and require validation in larger, more diverse populations. Increasing the number of participants and inclusion of other functional tasks (e.g. squats, single-leg stance) in future studies would address these limitations.

## Conclusions

This study provides evidence supporting the use of a three-repetition protocol for squat movement as analysed by the 3D MAI Motion markerless motion capture platform. Analysis demonstrated no statistically significant differences in mean kinematic measures and comparable variability between 3× and 5× conditions across all parameters. From a practical perspective, reducing repetition from five to three can improve time efficiency, reduce participant burden, and enhance protocol adherence. This may be particularly relevant in clinical populations, including elderly or frail individuals, where fatigue, discomfort, or compliance may limit feasibility. However, further validation in these groups is required. Accordingly, the 3× protocol may represent a practical alternative for routine testing.

## Figures and Tables

**Figure 5:**
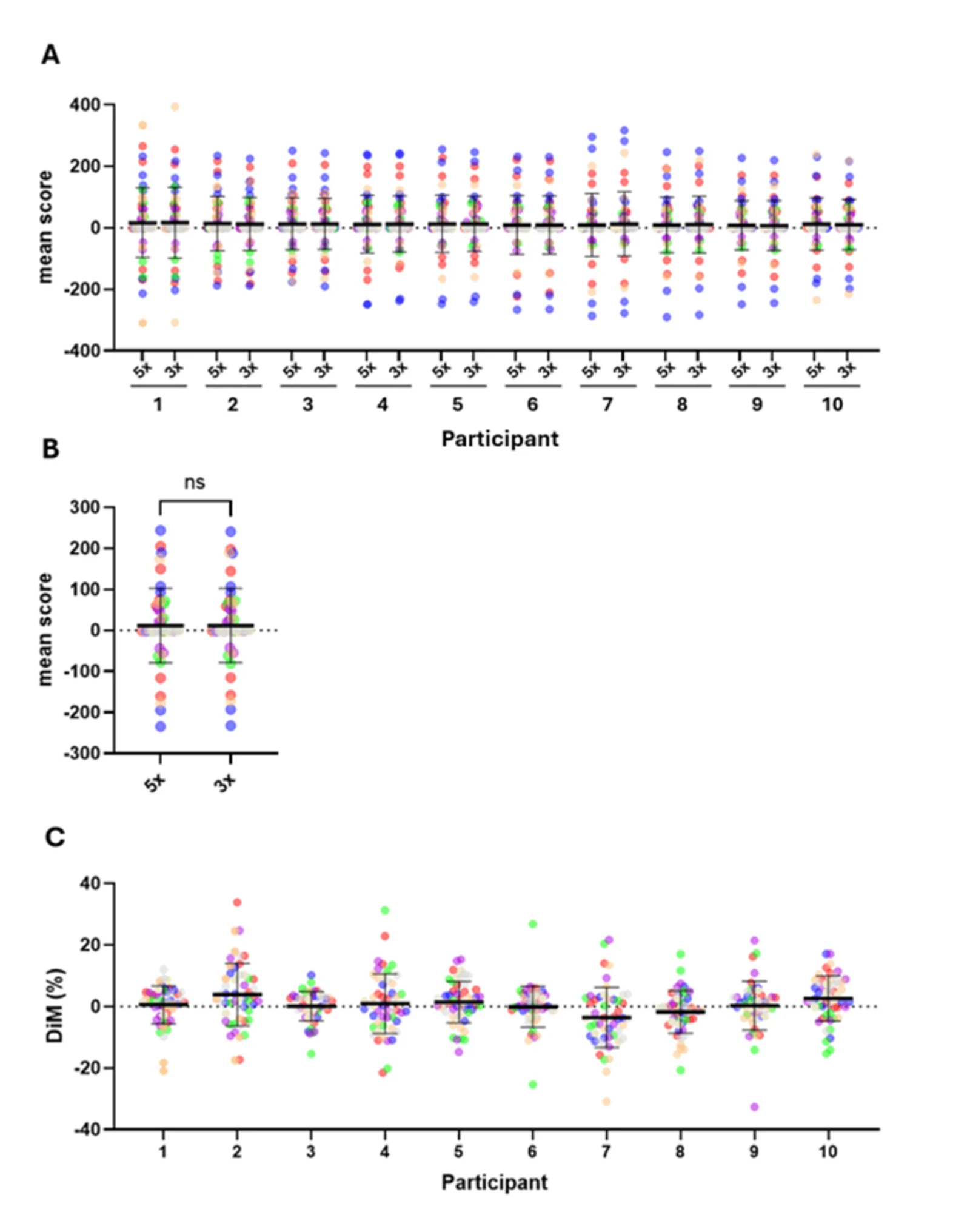
Difference in means (DiMs) derived from the analysis of 5× or 3× repeats of the squat test grouped by participant (A) The means for each parameter (describing knee angle (red), knee abduction (purple), hip angle (blue), hip abduction (orange), ankle angle (green), L/R movement (grey), and core movement (tan)) calculated from 5× or 3× repeats for each participant. (B) As in A following the calculation of the meta-means (derived by calculating the mean of the means for each participant for each parameter). (C) The DiMs between 5× and 3× for each parameter (presented as % of 5× mean plotted grouped by participant (1-10)). Mean±SD (black lines). No significant differences were observed between the means of any parameter comparing 5× vs. 3× (using ordinary one-way ANOVA followed by multiple comparisons or paired t-tests).
